# Ultrasensitive measurement of huntingtin protein in cerebrospinal fluid demonstrates increase with Huntington disease stage and decrease following brain huntingtin suppression

**DOI:** 10.1038/srep12166

**Published:** 2015-07-15

**Authors:** Amber L. Southwell, Stephen E.P. Smith, Tessa R. Davis, Nicholas S. Caron, Erika B. Villanueva, Yuanyun Xie, Jennifer A. Collins, Min Li Ye, Aaron Sturrock, Blair R. Leavitt, Adam G. Schrum, Michael R. Hayden

**Affiliations:** 1 Centre for Molecular Medicine and Therapeutics, Child and Family Research Institute, and Department of Medical Genetics, University of British Columbia, Vancouver, BC V5Z 4H4; Canada; 2 Mayo Clinic College of Medicine, Department of Immunology, Rochester, MN 55905.

## Abstract

Quantitation of huntingtin protein in the brain is needed, both as a marker of Huntington disease (HD) progression and for use in clinical gene silencing trials. Measurement of huntingtin in cerebrospinal fluid could be a biomarker of brain huntingtin, but traditional protein quantitation methods have failed to detect huntingtin in cerebrospinal fluid. Using micro-bead based immunoprecipitation and flow cytometry (IP-FCM), we have developed a highly sensitive mutant huntingtin detection assay. The sensitivity of huntingtin IP-FCM enables accurate detection of mutant huntingtin protein in the cerebrospinal fluid of HD patients and model mice, demonstrating that mutant huntingtin levels in cerebrospinal fluid reflect brain levels, increasing with disease stage and decreasing following brain huntingtin suppression. This technique has potential applications as a research tool and as a clinical biomarker.

Huntington disease (HD) is a fatal, inherited neurodegenerative disease that is uniquely caused by expansion of a polyglutamine encoding CAG trinucleotide repeat in the huntingtin (*HTT*) gene^1^. Although age of onset in HD can be predicted based on CAG repeat length^2^, this only accounts for 50–70% of the variation, while multiple known and unknown genetic and environmental factors account for the remainder^3^. Thus, there is a need for biomarkers that could more accurately predict disease onset or progression. Levels of mutant HTT (muHTT) protein in the brain could be such a biomarker. Additionally, there are multiple HTT lowering therapeutics in development, for which quantification of HTT levels in the brain would be useful to validate and quantify target engagement. There is significant evidence from murine studies that reducing brain levels of muHTT will provide benefit. In a conditional model of HD, it has been shown that turning off muHTT transgene expression results in significant functional recovery concomitant with clearance of accumulated muHTT^4^, indicating that HTT lowering therapies could not only halt or slow disease progression, but potentially reverse pathology. Pre-clinical trials of HTT lowering therapeutics in rodents have shown significant benefit in a broad range of HD-like phenotypes, even when administered post-symptomatically^5^,^6^,^7^,^8^,^9^,^10^,^11^,^12^,^13^, and some of these reagents are rapidly approaching clinical translation. A key question for these therapies is how can we determine if we have successfully reduced brain muHTT *in vivo*?

There is a critical need for a biomarker of brain HTT that can be measured in clinically accessible biofluids. Though HTT is predominately an intracellular protein, it is known to pass between and out of cells^14^,^15^. HTT protein in cerebrospinal fluid (CSF) may be brain derived, the result of secretion or release from injured or dying cells. CSF HTT levels may reflect brain HTT levels and provide a clinically useful biomarker. However, standard protein quantitation methods that have previously been applied to HTT detection in cell or tissue lysates, such as quantitative immunoblotting^16^,^17^, time-resolved fluorescence resonance energy transfer^17^,^18^, and Meso Scale electrochemiluminescence^19^, are not sufficiently sensitive to accurately detect and measure HTT protein in CSF.

To overcome this limitation, we have adapted the technique of microbead-based immunoprecipitation followed by flow cytometry (IP-FCM). IP-FCM is a highly sensitive method of protein detection that can be applied to either single proteins or those found in multiprotein complexes^20^,^21^. We have screened available anti-HTT antibodies and identified capture-probe pairs that accurately detect muHTT protein with enough sensitivity to allow measurement of muHTT protein in the CSF of HD patients and model mice. We have used muHTT IP-FCM to evaluate muHTT protein in CSF from control and premanifest and manifest HD gene positive individuals, as well as to evaluate the responsiveness of CSF muHTT to changes in brain HTT following antisense oligonucleotide (ASO)-mediated brain HTT suppression in HD model mice.

## Results

### HTT antibody screen and IP-FCM assay characterization

IP-FCM requires a pair of antibodies, a capture and a probe, that are simultaneously able to bind their protein target in its native state. Thus, we first performed a screen using 12 anti-HTT antibodies previously validated in assays performed in native conditions (described in Supplementary Table S1). Undiluted brain lysates from humanized Hu97/18 mice, which contain both human wt and muHTT^17^, were used. We measured the fluorescence signal obtained from each of the 144 possible capture/probe combinations (Supplementary Fig. S1). Two capture/probe antibody combinations stood out as excellent candidates, as they produced the highest fluorescence signals, MW1/BKP1 and HDB4E10/MW1. MW1 recognizes expanded polyglutamine^22^, BKP1 recognizes the N-terminus of HTT^23^, and HDB4E10 (hereafter HDB4) recognizes an epitope C-terminal to all known cleavage sites of HTT (aa1844–2131, supplier). The combination of HDB4 capture and MW1 probe produced an extremely strong fluorescence signal (10^5.5^), which suggested that this combination might be able to detect extremely low abundance HTT protein, as might be found in CSF.

**Figure 1:**
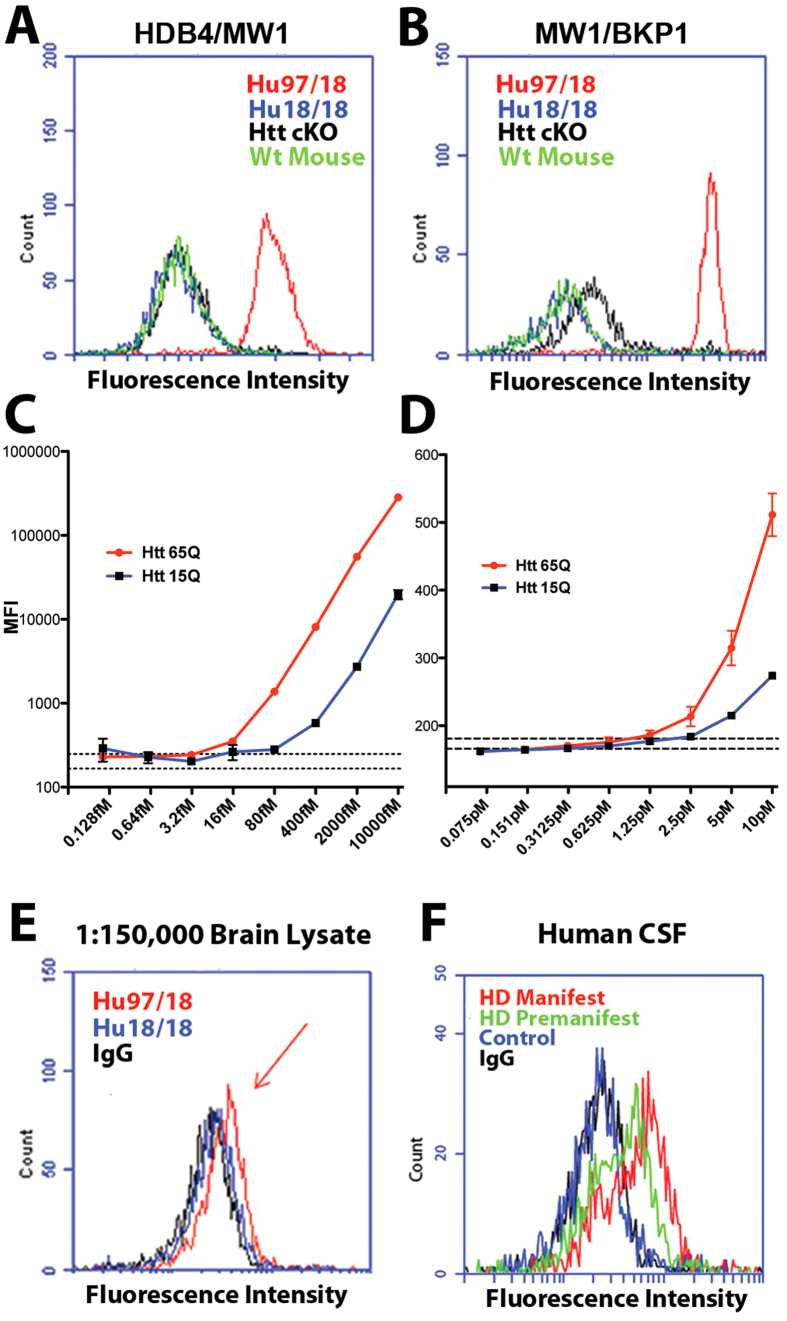
IP-FCM detects MuHTT in mouse brain and human CSF. (**a**,**b**) HTT IP-FCM in brain lysates from Hu97/18, Hu18/18, conditional Htt KO, and wildtype mice using (**a**) HDB4/MW1 or (**b**) MW1/BKP1. (**c**,**d**) HTT-IP-FCM median fluorescence intensity (MFI) using serial dilutions of recombinant HTT fusion protein with 15 or 65 Q in ACSF using (**c**) HDB4/MW1 or (**d**) MW1/BKP1. Dashed lines indicate the mean ± SEM of the no-protein controls, showing assay background. (**e**) HDB4/MW1 IP-FCM in 1:150,000 Hu97/18 or Hu18/18 brain lysate. Red arrow indicates positive signal. (**f**) HDB4/MW1 IP-FCM in CSF from premanifest (green) and manifest (red) HD gene positive individuals. Control CSF (blue) and no-CSF assay controls (black) are also shown.

We next tested the two antibody pairs for specificity. Constitutive knockout of the mouse HTT homolog is embryonic lethal^24^,^25^, so we established the specificity of the assays using Hu97/18 mice with human wt and muHTT and no mouse Htt (Hdh), Hu18/18 mice with human wtHTT and no mouse Htt, wildtype mice with mouse wtHtt, and conditional Htt knockout mice (cKO, generous gift of Dr. Paula Dietrich), with no Htt. Using IP-FCM with HDB4/MW1, we detected a strong signal in Hu97/18 mice, but not in wt, Hu18/18 or cKO mice (Fig. 1A). Similar results were obtained for MW1/BKP1 (Fig. 1B). Thus, we conclude that both antibody pairs specifically detect pathogenic muHTT, but not human wtHTT or mouse wtHtt.

Next, we performed a dilution experiment with recombinant protein to determine the sensitivity of the assays. A fusion protein consisting of HTT amino acids (aa) 1–171 (with 15 or 65 Q) and aa 1744–2234 (Supplementary Fig. S2) was produced in *E. coli* and purified using an N-terminal GST tag. The protein was brought to 1 nM in artificial cerebrospinal fluid (ACSF) or cKO brain lysate, serially diluted in 2- or 5-fold steps, and assayed using HBD4/MW1 or MW1/BKP1. As shown in Fig. 1C,D, in ACSF, HBD4/MW1 or MW1/BKP1produced median fluorescent intensities (MFI) that varied in a dose-dependent manner, with lower limits of detection approximately 16fM and 1.25 pM, respectively. Both assays preferentially recognize the expanded recombinant fusion protein, though at higher concentrations, they do both detect non-expanded recombinant HTT fusion protein. Conversely, in cKO brain lysate, both assays were specific for muHTT at all evaluated concentrations (Supplementary Fig. S3), which is consistent with the previous results in WT and HD model mouse brain lysate (Fig. 1A,B). Additionally, brain lysates from Hu97/18 mice were serially diluted in 5-fold steps to a final dilution of 1:150,000. At the final dilution, the MW1/BKP1 combination overlapped with the IgG control, but the HDB4/MW1 combination still provided signal significantly detectable above background MFI 341 ± 12 vs. 207 ± 8, N = 3, Fig. 1E). Thus, we have produced two IP-FCM assays that detect muHTT protein in CSF and brain lysate at very low abundance.

### Mutant HTT level in CSF of HD gene positive individuals increases with disease stage and correlates with clinical measures

**Figure 2:**
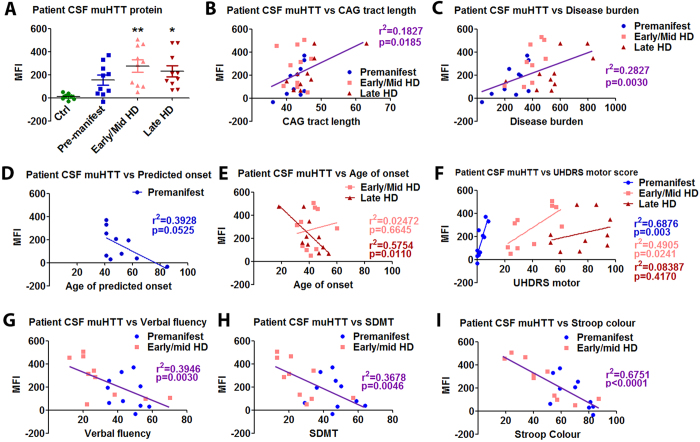
muHTT protein level in the CSF of HD mutation carriers increases with disease stage and is correlated to clinical measures. (**a**) HDB4/MW1 HTT IP-FCM was used to measure relative muHTT protein in CSF from control individuals, premanifest, early/mid HD, and late stage HD mutation carriers. In HD mutation carriers, CSF muHTT IP-FCM signal is significantly correlated with (**b**) CAG tract length, (**c**) disease burden (CAGn-35.5) X age), and (**d**,**e**) age of onset as well as with (**f**) UHDRS motor score in premanifest and early/mid HD but not in late stage HD mutation carriers. Additionally, significant correlations between (**g**) verbal fluency, (**h**) the symbol digit modality test (SDMT), and (I) the Stroop colour test of interference were observed in premanifest and early/mid HD. It was not possible to evaluate these cognitive measures in the late stage HD group.

HTT IP-FCM was used to measure HTT protein in CSF of control, premanifest HD mutation carriers, early/mid stage HD, and late stage HD individuals (Fig. 1F and Table 1). MW1/BKP1 did not detect a signal in control individuals or HD mutation carriers (data not shown). However, using the stronger HDB4/MW1 combination, muHTT protein was detected in the CSF of HD mutation carriers, but no signal above background was detected in control individuals (Fig. 2A). Significant variation in CSF muHTT protein level was observed in HD mutation carriers. While muHTT protein was detected in CSF of all manifest HD individuals, no signal above background was detected for some premanifest individuals, indicating the muHTT protein may not be a normal component of CSF or may be present in only minute quantities prior to significant pathology. In HD mutation carrier CSF, a significant correlation between muHTT IP-FCM signal and CAG tract length was observed (p = 0.0185, Fig. 2B), though this may be related to differences in antibody affinity rather than strictly to differences in muHTT protein level considering that an antibody recognizing the expanded polyglutamine tract is employed. A stronger correlation between muHTT IP-FCM signal and disease burden ((CAGn-35.5) X age) was observed (p = 0.0030, Fig. 2C), indicating an additional contribution of age to CSF muHTT signal, the likely result of increasing muHTT protein levels with age. In premanifest HD mutation carriers, there was a trend toward correlation between CSF muHTT IP-FCM signal and age of predicted onset as calculated by the Langbehn formula^2^ (Fig. 2D), which is dependent on CAG tract length. In manifest HD individuals, a strong correlation between CSF muHTT IP-FCM signal and age of onset was observed for late stage HD, and no significant correlation in these measures was observed for early/mid HD (Fig. 2E). These data indicate that at early stages there is a greater contribution of pathology and disease stage to CSF muHTT IP-FCM signal than that accounted for by CAG tract length, and that at late stages when pathology is very advanced in all individuals, the contribution of CAG tract length to IP-FCM signal accounts for a greater degree of individual variation. Thus, other factors not yet identified may influence individual variation in CSF muHTT IP-FCM signal.

Supporting this, CSF muHTT IP-FCM signal correlated with multiple clinical measures in premanifest and early/mid HD individuals. A strong correlation between CSF muHTT protein level and universal Huntington’s disease rating scale (UHDRS) motor score was observed in premanifest individuals (p = 0.003) and to a lesser degree in early/mid HD individuals (p = 0.0241), but not in late stage HD individuals (p = 0.4170) (Fig. 2F). We found that the premanifest individuals with CSF muHTT signal close to background were those with no or very low UHDRS motor scores, while those that had detectable muHTT protein in their CSF also had correlative sub-clinical UHDRS motor scores (Table 1). While cognitive performance could not be assessed in the late stage HD group, significant correlations were observed between multiple cognitive measures and CSF muHTT protein levels in premanifest and early/mid HD individuals, including verbal fluency, the symbol digit modality test (SDMT), and the Stroop colour test of interference (Fig. 2G–I). Taken together, these data provide strong evidence that levels of muHTT protein increase with increasing clinical impairment and that this relationship is particularly apparent in premanifest and early/mid HD. Thus, CSF muHTT protein as measured by IP-FCM could be a biomarker of HD onset and/or progression.

### CSF HTT levels respond to brain HTT suppression in mice

**Figure 3:**
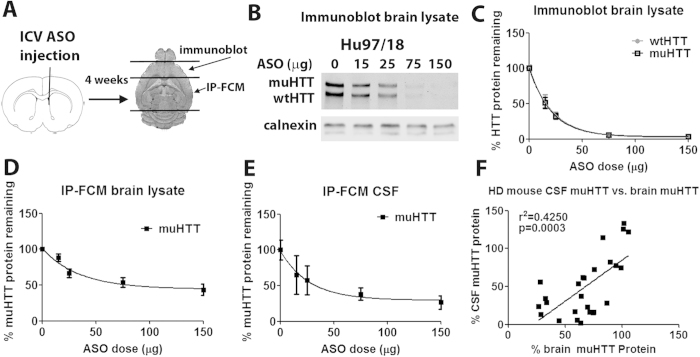
HTT protein in CSF is responsive to changes in brain HTT protein in HD model mice. (**a**) Hu97/18 mice were injected ICV with human HTT specific ASO. Four weeks later CSF and brains were collected. (**b**) Immunoblot of lysate of anterior forebrain with MAB2166 pan HTT antibody. (**c**) Quantitation by immunoblot in both sides of the brain from an N of 4–6 animals per dose. (**d**,**e**) IP-FCM measurement of relative HTT protein in lysate of (**d**) posterior forebrain and (**e**) CSF from the same animals. (**f**) Correlation between brain and CSF HTT levels in ASO treated Hu97/18 mice.

To investigate the effect of brain HTT lowering on CSF HTT levels, Hu97/18 mice were treated with a human HTT specific ASO expected to suppress both wt and muHTT protein (Fig. 3A). Four different doses of ASO or phosphate buffered saline (PBS) vehicle were delivered by intracerebroventricular (ICV) injection. Four weeks later, HTT protein was quantified in anterior forebrain lysates by allelic separation immunoblotting and normalized to PBS treated mice, demonstrating a dose dependent reduction of HTT protein in the brain (Fig. 3B,C). HTT IP-FCM in forebrain lysate from the same animals confirmed dose-dependent HTT lowering (Fig. 3D), though the actual muHTT KD in these samples is underestimated because the relationship between IP-FCM signal and muHTT protein concentration is exponential rather than linear (Fig. 1C,D). While greater inter-animal variation was seen in CSF of ASO treated mice, particularly at lower doses, a similar trend was observed (Fig. 3E). Comparison of brain and CSF HTT protein levels by IP-FCM demonstrates a significant correlation (p = 0.0003, Fig. 3F). These results demonstrate that reduction of brain HTT protein results in correlative reduction of CSF muHTT protein. Thus, CSF muHTT protein as measured by IP-FCM could be a much needed biomarker for brain muHTT reduction in clinical trials of HTT lowering therapeutics for HD.

### The brain is the major source of muHTT protein in the CSF

**Figure 4:**
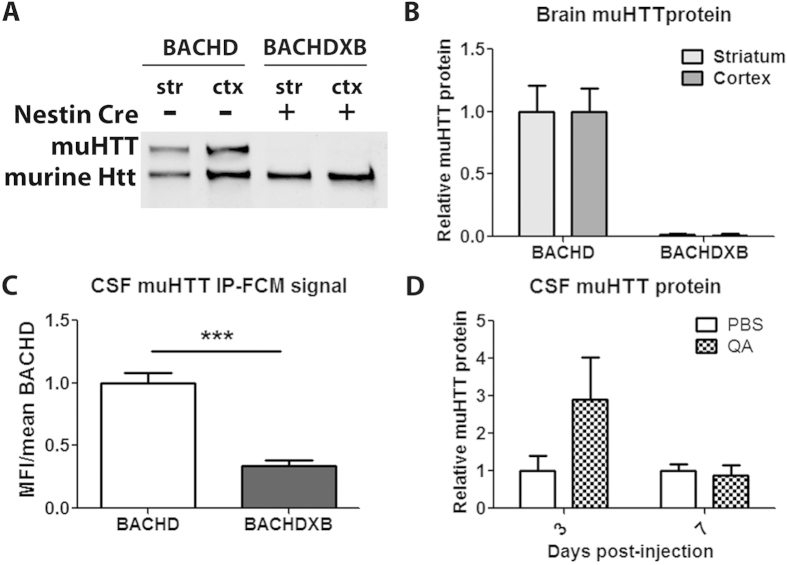
The brain is a major source of CSF muHTT protein. (**a**,**b**) BACHD and BACHDXB striatal and cortical tissues were evaluated for HTT protein levels by immunoblot and quantified in an N of 4 animals per group. (**c**) CSF HTT IP-FCM from the same animals demonstrates a 66% reduction in IP-FCM signal when brain muHTT protein is absent. (**d**) Quantification in an N of 4 animals per group of relative muHTT protein in CSF of Hu97/18 mice 3 or 7 days following intrastriatal injection of PBS vehicle or QA neurotoxin.

The response of CSF muHTT protein levels to brain HTT suppression indicates that the brain is a source of CSF muHTT protein. In addition, the lack of muHTT protein signal in CSF of some premanifest HD mutation carriers suggests that muHTT protein may not be a normal component of CSF and that it may be released into CSF in a disease state. To further investigate the source of muHTT protein in the CSF, we used BACHD mice^26^, which express full-length human muHTT with loxP sites surrounding exon 1. By crossing BACHD mice with mice expressing rat nestin cre in the central and peripheral nervous system^27^, we generated BACHD mice with muHTT specifically knocked out of the brain and nervous tissue (BACHDXB). Immunoblot of brain tissues from BACHD and BACHDXB mice shows a complete lack of muHTT protein in striatum and cortex of BACHDXB mice (Fig. 4A,B). Evaluation of muHTT protein in CSF by IP-FCM demonstrates a 66% loss of CSF muHTT IP-FCM signal in BACHDXB mice as compared to BACHD mice (Fig. 4C). Because the relationship between IP-FCM signal and muHTT protein concentration is exponential (Fig. 1C,D), this represents a greater than 66% reduction in CSF muHTT protein level, indicating that the brain is the major source of muHTT protein in the CSF. Additionally, we observed a strong trend toward a transient increase in CSF muHTT protein in Hu97/18 CSF following striatal injection of the neurotoxin quinolinic acid (QA) (Fig. 4D). Three days following QA injection, when cell death is occurring, the mean CSF muHTT protein level was 3 fold higher than the mean level for PBS vehicle injected mice. This effect failed to reach significance due to high inter animal variability, which is likely due to surgical and neurodegenerative variation. At seven days following QA injection, when the cell death process is predominately completed and lesions are fully developed, CSF muHTT protein levels were similar in PBS and QA injected animals. These data indicate that active cell death leads to release of muHTT protein into the CSF, which is fairly rapidly cleared. The dynamic nature of this system and the rapid response to CNS changes make CSF muHTT IP-FCM a valuable biomarker of brain muHTT protein.

## Discussion

As the fundamental cause of HD, reducing levels of muHTT protein in the brain has substantial therapeutic potential, and a number of HTT lowering therapies are currently in pre-clinical and clinical development. Clinical trials of HTT lowering therapies will require biomarkers of brain HTT levels in order to validate and quantify target engagement and efficacy and to inform appropriate dosing. The HTT IP-FCM assays developed here represent essential tools for this purpose.

Moreover, these assays are valuable research tools that can be used to investigate features of HD and the HTT protein. For instance, the two assays developed here, though both specific for muHTT protein, likely detect different pools of muHTT. With MW1 binding in exon 1 and HDB4 binding at an epitope C-terminal to all known cleavage sites, this assay likely detects full-length muHTT protein. The BKP1/MW1 assay, however, employs two antibodies binding in exon 1 of HTT, indicating that this assay likely detects both full-length and N-terminal fragments of muHTT, which are known to be pathogenic and enriched in HD striatum^28^,^29^. It is conceivable that these two assays together could be used to investigate the natural history of HTT proteolysis in the progression of HD.

In this study, we have used HTT IP-FCM to determine that muHTT protein increases in the CSF of HD mutation carriers with disease stage. CAG tract length correlates with CSF muHTT level in HD mutation carriers, as do the clinical measures disease burden and age of onset, both of which are influenced by CAG tract length.

We also identified correlations between CSF muHTT IP-FCM signal and multiple motor and cognitive clinical measures that are independent of CAG tract length, indicating a significant contribution of disease to CSF muHTT protein level. Additionally, in some premanifest HD mutation carriers, no detectable muHTT protein was observed in CSF. Interestingly, we found that premanifest individuals that lacked muHTT protein in CSF were also those that did not score on the UHDRS motor test, while those that did have muHTT in CSF also had sub-clinical motor deficits. This reflects the contribution of disease to CSF muHTT protein level irrespective of CAG tract length. For example, subjects 31 and 41 both have 45 CAG and were collected at 39 years of age, yielding identical disease burdens and predicted onsets. However, subject 31 has a low UHDRS motor score of 2 and a low CSF HTT signal (MFI 63), while subject 41 has a high sub-clinical UHDRS motor score of 8 and a high CSF HTT signal (MFI 332). Taken together, these results suggest that muHTT protein may not be a normal component of CSF or is only present in minute amounts, but is released to the CSF in a disease state, possibly as a result of injured or dying cells. This hypothesis is supported by our finding that QA injection induces a transient increase in CSF muHTT protein levels. Overall, we find that CSF muHTT IP-FCM signal varies with multiple factors including CAG tract length, disease burden, disease stage, motor and cognitive scores, and other unknown factors. However, for longitudinal studies within the same patients, the contribution of CAG tract length and other non-disease related factors to CSF muHTT IP-FCM signal would be the same in baseline and subsequent samples, allowing for accurate relative quantitation. Thus, HTT IP-FCM in CSF could provide a biomarker of HD onset.

Additionally, using HTT IP-FCM, we have found that brain HTT suppression results in reduction of muHTT protein in CSF of HD model mice, and that CSF muHTT levels are correlated with brain muHTT levels in ASO treated animals. Moreover, we find that knocking out muHTT selectively in the nervous system of BACHD mice results in a dramatic reduction of CSF muHTT protein. These results indicate that the brain is a major contributor, if not the source, of CSF muHTT protein, and that muHTT IP-FCM in CSF could provide a critically needed pharmacodynamic and target engagement biomarker for upcoming HTT lowering clinical trials.

These findings also have far-reaching implications for a wide range of CNS indications, including idiopathic diseases for which IP-FCM CSF biomarkers could be identified as predictors of disease onset in at risk individuals or as biomarkers in therapeutic trials.

## Methods

### HTT antibodies

BKP1, HD46, and HD650 antibodies^23^,^30^ were generated in the Hayden lab. MW1 and MW7 antibodies^22^, generated in Paul Patterson’s lab, were obtained through the Developmental Hybridoma Bank. All other antibodies were commercially obtained: 2166, 2168, 1C2 (Millipore), HDB4E10, HDA3E10, EP867Y (Abcam), H-300 (Santa Cruz).

### Human sample collection and preparation

All human samples were collected with an approved protocol and in accordance with the guidelines of the institutional review board of the University of British Columbia and the full informed consent of the subjects. Cerebrospinal fluid from HD gene positive and control individuals was collected at the University of British Columbia’s Centre for Huntington Disease. Thirty subjects with the HD gene mutation and 7 control subjects, age-matched and lacking the HD mutation, were recruited (see Table 1). CSF was obtained by lumbar puncture, examined by microscopy, and centrifuged to remove cells. The acellular supernatant was aliquoted and frozen at −80 °C. There was no significant contamination of CSF by blood cells (median erythrocyte count 0.5 × 10^6^/L, range 0–171; median leukocyte count 1.0 × 10^6^/L, range 0–17).

### Mice, treatments, and sample preparation

Hu97/18^17^ and BACHD^26^ HD model mice were maintained under a 12 h light:12 h dark cycle in a clean facility and given free access to food and water. Experiments were performed with an approved protocol and in accordance with the guidelines of the animal care committee of the University of British Columbia. For ASO treatment, a fully phosphorothioate modified gapmer ASO with 5 locked nucleic acid (LNA) modifications in each wing, matching a sequence in intron 22 of the *HTT* gene (5’-TAATACGTAAGTGTCACAA-3’, custom synthesized by Exiqon) was delivered by intracerebroventricular (ICV) injection as in^31^ at doses ranging from 15 μg to 150 μg and diluted to a total volume of 10 μl in sterile phosphate buffered saline (PBS). Brains and CSF samples were collected four weeks post-surgery. For QA treatment, 25 nM QA in PBS vehicle was delivered by unilateral intrastriatal injection as in^32^. Brains and CSF samples were collected 3 or 7 days post-surgery. For collection of treated samples and tissues, mice were anesthetized with avertin and CSF was collected through the cisterna magna, briefly centrifuged, and pipetted into a pre-chilled tube. The brain was then removed and placed on ice for 1 min to increase tissue rigidity. Brains were then placed in a 1 mm rodent brain matrix (ASI instruments), and the second most anterior 2 mm section was collected, divided into hemispheres, and placed in pre-chilled tubes. The remaining forebrain was divided into hemispheres and placed into pre-chilled tubes. All tissue and CSF samples were immediately snap frozen and stored at −80 °C until use.

### HTT quantitation by immunoblot

Brain HTT protein was quantified by allelic separation immunoblotting as in^16^. Briefly, the right and left 2 mm anterior brain sections from each animal were homogenized in SDP buffer (50 mM Tris pH8.0, 150 mM NaCl, 1% Igepal, 40 mM B-glycerophosphate, 10 mM NaF, 1X Roche complete protease inhibitor, 1 mM sodium orthovanadate and 800 mM PMSF), and 40 μg total protein was resolved on 10% low-bis acrylamide gels (200:1 acrylamide:bis). Proteins were transferred to 0.45 μm nitrocellulose membranes, which were probed for HTT (MAB2166 1:2000, Millipore) and calnexin as a loading control (1:10,000, Sigma). Secondary IR dye 800CW goat anti-mouse (1:250, Rockland) and AlexaFluor 680 goat anti-rabbit (1:250, Molecular probes) antibodies and the LiCor Odyssey infrared imaging system were used to visualize proteins. For ASO treatment, the intensity of each allele of HTT was normalized to calnexin and then to the mean value for the same allele from PBS injected animals on the same membrane. For BACHD and BACHDXB mice, the intensity of muHTT was normalized to endogenous murine Htt from the same sample and then to the mean value for BACHD mice for the same brain region.

### Generation of recombinant HTT fusion proteins

For the calibration curves, a recombinant Htt fusion protein was generated that contained the antibody binding sites for MW1, BKP1 (both in the N terminal region) and HDB4 (aa1844–2131) (Supplementary Fig. S2). Recombinant huntingtin 1–171/1744–2234 fusion proteins with 15 or 65 glutamines were synthesized by performing PCR of full length HTT (with 15 or 65 CAG repeats) templates using primers F1f 5’ GAT CGG ATC CAT GGC GAC CCT GGA AAA G 3’, F1r 5’ GAT CGC GGC CGC GTC TAA CAC AAT TTC A, F2f 5’ GATC CTC GAG AGG TTT CTA TTA CAA CTG GTT G 3’ and F2r 5’GATC GCGGCCGC GAC CAC CAC CAG GTA CTG TGC 3’ to generate two amplicons, F1 and F2. Following purification with the QiaQuick PCR purification kit (Qiagen), F1 amplicons were digested with BamHI and XhoI, F2 amplicons were digested with XhoI and NotI and the pGEX-6p-1 expression vector was digested using BamHI and NotI overnight at 37 °C. Digests were then run on a 1% agarose-TBE gel, bands of interest excised and purified using the QiaQuick Gel Extraction kit (Qiagen). Ligation of digested pGEX-6p-1, F1 and F2 was done at room temperature for 1 hour, then transformed into DH5 cells and plated overnight on LB-agar plates containing 100 μg/mL ampicillin. Clones were screened using a colony PCR with primers F1f and F2r. Positive clones were amplified overnight in LB and purified using a mini-prep kit (Qiagen). Constructs were sequenced to confirm identity and correct frame of fusions. Subsequently, constructs were transformed into BL21 DE3 cells and individual colonies were amplified overnight in 5 mL LB cultures with 100 μg/mL ampicillin. 16 hours after inoculation, cultures were transferred to 200 mL of fresh LB and grown for ∼2 hours until OD 600 values reached 0.8. Cultures were then induced using 0.2 mM IPTG for 2.5 hours at 37 °C. Cultures were lysed and purified using the GST tag on a column and eluted using 20 mM glutathione in TBS (50 mM Tris, 150 mM NaCl pH 7.5). Eluates were buffer-exchanged into PBS using Amicon Ultra 10K MWCO centrifugal filters (Millipore). Protein was quantified using a BCA assay (Pierce) and checked for purity using silver-stained SDS-PAGE gels. Protein concentration in mg/ml was converted to mol/L using the predicted molecular weight of the amino acid sequences, and samples were diluted to 1 nM in ACSF (in MM: NaCl 125, KCl 2.5, NaH_2_PO_4_ 1.25, MgCl_2_ 1, NaHCO_3_ 26, CaCl_2_ 2 Dextrose 25). A dilution series was made and IP-FCM was performed (see below).

### IP-FCM

The IP-FCM technique has been previously described^20^,^21^,^33^. Briefly, capture antibodies were coupled to 5 um CML latex microbeads (Invitrogen) and counted on a hemocytometer before storage at 4 °C. Probe antibodies were biotinylated using EZ-Link Sulfo-NHS-Biotin (Thermo Scientific), free biotin removed by buffer exchange in Amicon Ultra 3K MWCO spin columns (Millipore), and antibody concentration brought to 0.5 mg/ml before storage at 4 °C in PBS. Brain samples were lysed for 20 minutes on ice in NP40 buffer (150 mM NaCl, 50 mM Tris pH 7.4, Halt Phosphatase and Protease inhibitors (Pierce), 2 mM Sodium Orthovanadate, 10 mM NaF, 10 mM Iodoacetamide, 1% NP40) and cleared by centrifugation. Cleared brain samples were diluted 1:50 in NP40 buffer unless otherwise stated. Approximately 10^4^ beads in 5 ul NP40 buffer were mixed with either 25 ul of recombinant protein in ACSF, or 50 ul of brain lysate, or 50 ul of human CSF, or 10 ul of mouse CSF and left to immunoprecipitate HTT overnight at 4 °C with rotation to prevent beads settling out of suspension. Beads were then washed in IP-FCM buffer (100 mM NaCl, 50 mM Tris pH 7.4, 1% Bovine Serum Albumin (Sigma), 0.01% Sodium Azide) and incubated with biotinylated probe antibodies for two hours, followed by another wash in IP-FCM buffer, incubation with 1:200 Streptavidin-PE (BD Biosciences) for one hour, a final wash, and measurement on an Accuri flow cytometer (BD Biosciences). Bead doublets were gated out based on forward scatter area vs. forward scatter height plots, and a singlet bead gate was defined based on forward scatter height vs. side scatter height. All samples were run in duplicate (except mouse CSF, of which only ∼10 ul was collected), and the average of the median fluorescence intensity in the FL2 channel in the singlet bead gate indicated the abundance of HTT in the sample.

### Statistical analysis

Differences between sample means were analyzed by two-tailed Student’s T-tests, and one or two-way ANOVAs were used for multiple comparisons, indicated in the text. Correlations were analyzed by linear regression with r^2^ and p values indicated on graphs. P values of less than 0.05 were considered statistically significant. N’s represent individual mice or human samples. All IP-FCM samples, except for mouse CSF samples where sufficient volume was not available, were run in duplicate and averaged to obtain the single value for each sample.

## Additional Information

**How to cite this article**: Southwell, A. L. *et al.* Ultrasensitive measurement of huntingtin protein in cerebrospinal fluid demonstrates increase with Huntington disease stage and decrease following brain huntingtin suppression. *Sci. Rep.* **5**, 12166; doi: 10.1038/srep12166 (2015).

## Supplementary Material

Supplementary Information

## Figures and Tables

**Table 1:** Clinical information for human CSF samples.

Subject ID	HD status	Sex	Age	CAG size	Disease burden	Age of onset	UHDRS motor	Verbal fluency	SDMT	Stroop colour	MFI
13	Control	Female	29	18			0	32	53	80	−5.00
21	Control	Male	55	17			0	28	41	78	17.00
23	Control	Female	47	17			0	39	57	100	−9.00
27	Control	Female	66	21			0	38	48	80	13.50
28	Control	Female	38	20			0	45	44	73	49.00
29	Control	Female	58	17			0	48	60	88	38.00
30	Control	Male	27	17			0	55	56	88	−29.00
31	premanifest	Male	39	45	370.5	41*	2	35	29	52	63.00
32	premanifest	Female	33	44	280.5	44*	0	58	48	80	31.00
33	premanifest	Male	30	42	195	52*	0	43	59	81	77.00
35	premanifest	Male	57	41	313.5	57*	5	34	36	59	193.00
37	premanifest	Male	27	44	229.5	41*	1	35	47	72	255.00
39	premanifest	Female	68	36	34	85*	0	50	45	83	−35.00
41	premanifest	Female	39	45	370.5	41*	8	42	38	54	332.00
42	premanifest	Female	38	45	361	41*	6	49	45	59	369.50
48	premanifest	Male	40	43	300	48*	4	53	50	70	207.00
49	premanifest	Female	23	40	103.5	63*	1	53	64	83	40.00
15	Early	Female	43	47	494.5	34	29	26	34	50	341.50
18	Early	Female	56	39	196	45	22	70	57	87	107.50
19	Early	Male	44	46	462	41	24	22	30	70	52.00
25	Early	Male	42	44	357	31	27	23	20	40	314.50
16	Mid	Male	72	41	396	60	61	27	17	40	285.00
17	Mid	Male	51	45	484.5	43	54	20	13	24	505.50
20	Mid	Male	51	43	382.5	44	54	20	21	34	465.50
22	Mid	Male	53	43	397.5	35	41	38	26	55	15.00
24	Mid	Female	45	43	337.5	38	29	56	33	57	97.50
26	Mid	Male	56	37	84	46	57	12	13	19	455.00
34	Late	Male	53	46	556.5	38	53				213.00
36	Late	Female	46	47	529	35	78				164.00
38	Late	Male	63	44	535.5	54	60				67.00
40	Late	Female	46	40	207	39	53				147.00
43	Late	Male	66	44	561	50	94				123.00
44	Late	Female	67	48	837.5	40	96				346.50
45	Late	Female	48	48	600	36	86				474.00
46	Late	Female	60	43	450	47	94				209.00
47	Late	Male	66	42	429	47	74				71.00
50	Late	Male	29	63	797.5	18	72				474.00

MFI-median fluorescence intensity, SDMT-symbol digit modality test, UHDRS-universal Huntington disease rating scale.

^*^predicted^2^.
