# Supplementary material for: Ultrasensitive measurement of huntingtin protein in cerebrospinal fluid demonstrates increase with Huntington disease stage and decrease following brain huntingtin suppression
Source: Sci Rep. 2015 Jul 15;5:12166. doi: 10.1038/srep12166 (PMC4502413; doi:10.1038/srep12166)
Supplement: Supplementary Information [file srep12166-s1.pdf]

Supplementary materials for: Ultrasensitive measurement of huntingtin protein in cerebrospinal fluid demonstrates increase with Huntington disease stage and decrease following brain huntingtin suppression

Amber L. Southwell, Stephen E.P. Smith, Tessa R. Davis, Nicholas S. Caron, Erika B. Villanueva, Yuanyun Xie, Jennifer A. Collins, Min Li Ye, Aaron Sturrock, Blair R. Leavitt, Adam G. Schrum, Michael R. Hayden

Supplementary Table S1. Summary of anti-HTT antibodies screened

Antibody	Host	Species specificity	Epitope	HTT specificity	Validated applications	Source
HD46	Mouse	Hu,Ms	aa1-17	N-term	WB,IF,IP	Michael Hayden
BKP1	Rabbit	Hu,Ms	aa1-17	N-term	WB,IF,IP,FRET	Michael Hayden
HD650	Mouse	Hu	aa650-655	pan	WB,IF,IP	Michael Hayden
MW1	Mouse	Hu,Ms	polyQ	mutant	WB,IF FRET	Paul Patterson
MW7	Mouse	Hu,Ms	polyP	pan	WB,IF	Paul Patterson
2166	Mouse	Hu,Ro,Rb	aa181-810	pan	WB,IF,IP,ELISA,FRET	Commercial
2168	Mouse	Hu,NHP	aa2146-2541	C-term	WB,IF,IP,ELISA	Commercial
1C2	Mouse	Hu	polyQ	mutant	WB,IF,IP	Commercial
HDB4E10	Mouse	Hu,Ms,Rb	aa1844-2131	C-term	WB,IF,IP,ELISA	Commercial
HDA3E10	Mouse	Hu,Ms,Ra	aa997-1276	pan	WB,IF,IP,ELISA	Commercial
EP867Y	Rabbit	Hu,Ro	aa511-588	pan	WB,IF,IP,ELISA,FACS	Commercial
H-300	Rabbit	Hu,Ro	aa2845-3144	C-term	WB,IF,IP,ELISA	Commercial

Hu-human, Ms-mouse, Ro-rodent, Rb-rabbit, NHP-non-human primate, Ra-rat, WB-Western blot, IF-immunofluorescence, IP-immunoprecipitation, FRET-fluorescence resonance energy transfer, ELISA-enzyme-linked immunosorbent assay, FACS-fluorescence activated cell sorting

Supplementary Figure S1

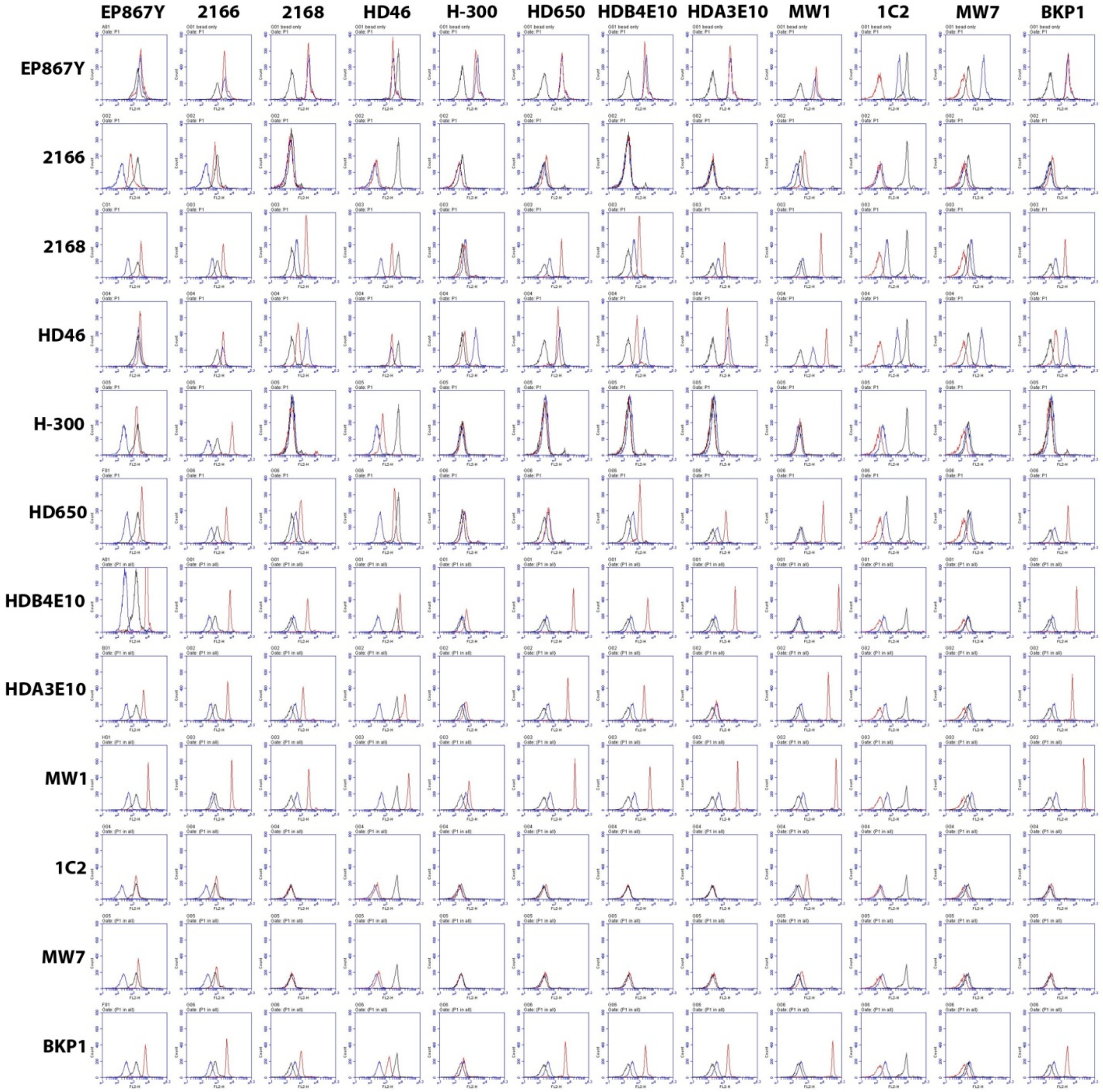

Supplementary Figure S1. Screen to identify anti-HTT capture-probe antibody pairs for IP-FCM. Brain lysate from Hu9718 mice containing abundant human wt and muHTT protein was immunoprecipitated onto latex micro-beads and detected by flow cytometry. Rows represent capture antibodies. Columns represent probe antibodies. Black and blue traces are IgG capture control and IgG probe control, respectively. Red traces are experimental. Red traces of higher magnitude (i.e. shifted to the right) than control traces indicate positive HTT IP-FCM signal.

Supplementary figure S2

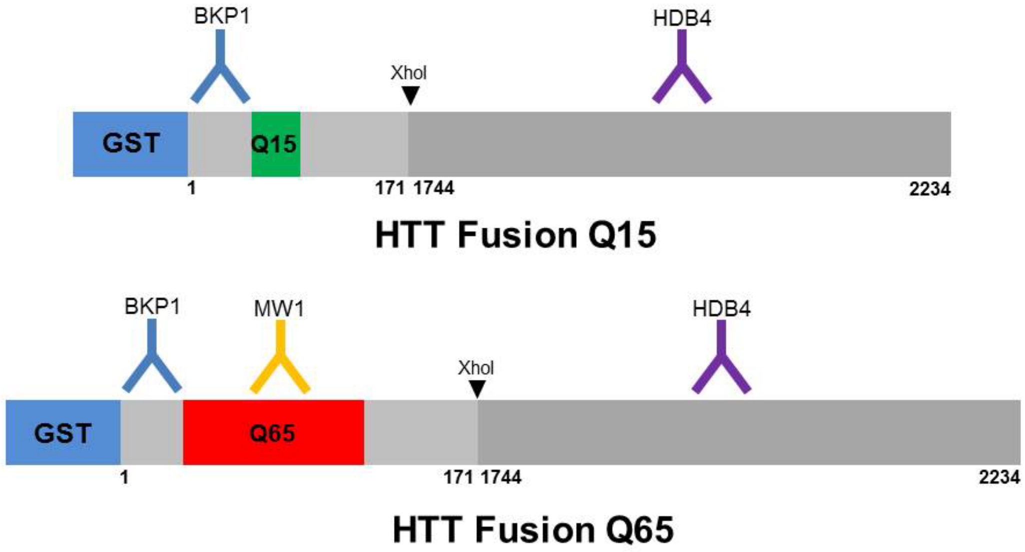

Supplementary figure S2. Schematic of recombinant HTT fusion proteins. Recombinant HTT fusion protein with either 15 (non-expanded) or 65 (expanded) glutamines was generated from the N-terminal 171 aa, containing the BKP1 and MW1 binding sites, and aa 1744-2234, which encompasses the antigenic fragment that was used to generate HDB4, aa 1844-2131. The XhoI restriction site found naturally in HTT at residue 171 was used to generate the fusion protein.

Supplementary figure S3

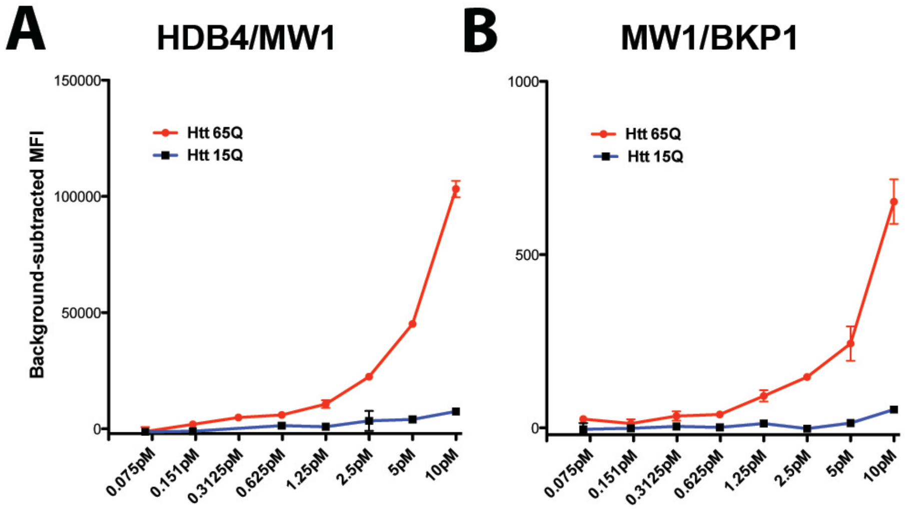

Supplementary figure S3. Standard curve of recombinant HTT fusion protein in cKO brain lysate. (a,b) HTT-IP-FCM using serial 1:50 dilutions of recombinant HTT fusion protein with 15 or 65 Q using (a) HDB4/MW1 or (b) MW1/BKP1 in cKO brain lysate. Dashed lines indicate the mean ± SEM of the no-protein controls, showing assay background.
